# Supplementary material for: Rapid turnover of life-cycle-related genes in the brown algae
Source: Genome Biol. 2019 Feb 14;20:35. doi: 10.1186/s13059-019-1630-6 (PMC6374913; doi:10.1186/s13059-019-1630-6)
Supplement: Supplementary file 3 — Protein alignments, corresponding Gblocks html file and CDS sequences for the lineage conserved gametophyte-biased genes. (ZIP 2109 kb) [file 13059_2019_1630_MOESM3_ESM.zip › Con_GA_alignments/Ec-18_000920.aa_ali.fasta-gb.htm]

Ec-18\_000920.aa.fasta.trans.aa\_ali.fasta


## Gblocks 0.91b Results

Processed file: **Ec-18\_000920.aa.fasta.trans.aa\_ali.fasta**  
Number of sequences: **2**  
Alignment assumed to be: **Protein**  
New number of positions: **354** (selected positions are underlined in blue)

```
                         10        20        30        40        50        60
                 =========+=========+=========+=========+=========+=========+
Ec-18_000920     ------------------------------------------------------------
MSTRG.27118.1    MPDIDDGGGTQPPETQLPLPSIGGSSHGTTGKGEPSDDPGNDVLAGAAGGEGDGDSLRLS
                                                                             


                         70        80        90       100       110       120
                 =========+=========+=========+=========+=========+=========+
Ec-18_000920     ------------------------------------------------------------
MSTRG.27118.1    IDSDLDDTWVADGPKGPTKKKRTRFGAIGEWIDDYKTRKSGYGPPYYAEYGGDLPGDCNR
                                                                             


                        130       140       150       160       170       180
                 =========+=========+=========+=========+=========+=========+
Ec-18_000920     MGADLIDVDELLRNGANPNLGHREDQGLTPIHFAARFGNVELLKMLKRAGGDPGITNALG
MSTRG.27118.1    MGADLLEIDELLRNGANPNVGHQEDQGLTPLHFAARFGNVELLKMLKRAGGDPNVTNAVG
                 ############################################################


                        190       200       210       220       230       240
                 =========+=========+=========+=========+=========+=========+
Ec-18_000920     LTPLSYTCAFKLSLSKNGDDSWERQQVAAIKWLLGQGANPNHVDKAGYTPLAWACRSGIF
MSTRG.27118.1    LTPLSYSCAFKLSLSAKGGDSAEGKHVAAVEWLLDQGANPNHVDKAGYTPLAWACRSGVY
                 ############################################################


                        250       260       270       280       290       300
                 =========+=========+=========+=========+=========+=========+
Ec-18_000920     PAVVALVEAGADVMQRRPELPGGSHVRLPKADPLAVTTVKQIERYVQNKINTILLREGAL
MSTRG.27118.1    LIVVALVDAGADVMQRRPNLPGKSQVCLPKADPLAVTTDKKIERYVQAKINAILQREAAM
                 ############################################################


                        310       320       330       340       350       360
                 =========+=========+=========+=========+=========+=========+
Ec-18_000920     LRRECKVDLRSRRLKEIQAAIARKKEARAFVIQEKQRMKQTEELSLELMKVDKAEEARKA
MSTRG.27118.1    ICREVKVDCRTRRLKEIQEAIAKKKEARLFIIQEKQRVKETEELSFELMKADKAEEARRA
                 ############################################################


                        370       380       390       400       410       420
                 =========+=========+=========+=========+=========+=========+
Ec-18_000920     RRDWLRLMKVSPGIWVKVEG-------------AASAGTPG--VRARKPRPRWVFDTSTA
MSTRG.27118.1    RRDWLRLMKVSPGIWAKVEAGAEGLSPTSEEGRGQSSLSPGSPASKRKPRPRWIFDTSTA
                 ###################                           ##############


                        430       440       450       460       470       480
                 =========+=========+=========+=========+=========+=========+
Ec-18_000920     VSKSEAKAHGVLEEARQVEAFIINGGQKQKILAPRWRSMTGKRLLRLEHGQRVGLAAKVK
MSTRG.27118.1    VSKKEAAAHDVLEEARQVETFILNGVQKQKILAPRWRAMTGKRLLRLEHGKRLGAAAKGK
                 ############################################################


                        490       500       510       520       530
                 =========+=========+=========+=========+=========+=
Ec-18_000920     GRRRDRQGDGSRGGGVGGDAESGSGPPPPLSGLEEEGSDTSSSSGGSEEGN
MSTRG.27118.1    DRHRHT-----RE---AGDNVSGGG--APLSGLEEEGSETSSSSG-SDNGR
                 ####                        #################
```

```
Parameters used
Minimum Number Of Sequences For A Conserved Position: 2
Minimum Number Of Sequences For A Flanking Position: 2
Maximum Number Of Contiguous Nonconserved Positions: 8
Minimum Length Of A Block: 10
Allowed Gap Positions: None
Use Similarity Matrices: Yes
```

```
Flank positions of the 3 selected block(s)
Flanks: [121  379]  [407  484]  [509  525]  

New number of positions in Ec-18_000920.aa.fasta.trans.aa_ali.fasta-gb:  354  (66% of the original 531 positions)
```
